# How to protect Chinese medicine prescription more effectively?

**DOI:** 10.3389/fphar.2025.1545986

**Published:** 2025-04-25

**Authors:** Xiaochang Liu, Gaoyuan Zhai

**Affiliations:** ^1^ School of Law, Tianjin University, Tianjin, China; ^2^ School of Law, Tianjin University, Tianjin, China

**Keywords:** TCM prescriptions, intellectual property, law of the People’s Republic of China on the traditional Chinese medicine, active measures, passive defenses

## Abstract

As an important carrier of traditional Chinese medicine (TCM) knowledge, TCM prescriptions is the crystallization of the wisdom of Chinese medical practitioners through the ages, and occupies a pivotal position in the traditional knowledge system of Chinese medicine in China. However, under the current legal framework, despite the establishment of intellectual property rights system and the adoption of the Law of the People’s Republic of China on Traditional Chinese Medicine, which provide a certain legal foundation for the protection of TCM, these systems still face difficulties in providing adequate and effective protection for TCM prescriptions in practical operations. Based on this, this paper will adopt the comparative research method and literature analysis method to deeply explore advanced systems in other countries for the protection of traditional medicine knowledge and to identify the shortcomings and deficiencies of the current systems in China. On this foundation, the paper will focus on discussing how to improve and upgrade China’ TCM prescription protection system through both active improvement measures and passive defence measures. The goal is to prevent improper utilization of China’s TCM prescriptions by other countries while also facilitating China’s TCM enterprises to enter the international arena, enhancing China’s national influence and voice in the field of TCM, and contributing wisdom and strength to the inheritance and development of the TCM industry.

## 1 Introduction

The traditional knowledge of Chinese medicine, as a treasure of China’s excellent traditional culture, contains immense value. When the SARS epidemic ravaged the world in 2003, Chinese medicine played a pivotal role in treatment due to its unique effectiveness (Zeng and Liu, 2011). Similarly, in the recently concluded COVID-19 pandemic, the preventive and therapeutic effects of Chinese medicine were once again fully verified and affirmed. (The Experience of Tradition Chinese, 2024) TCM Prescriptions, as a core component of the traditional knowledge system of Chinese medicine, are undeniably significant. However, the current legal system in China cannot provide sufficient and effective legal protection. Therefore, this article will first discuss the classification of TCM prescriptions and their unique nature. Taking this as a starting point, this article will explore the institutional designs and practical experiences of typical countries in the protection of traditional medicine. At the same time, this article will also carefully analyze the specific issues and challenges faced by China’s current legal system in the protection of TCM prescriptions. On this basis, the article will put forward targeted suggestions for improvement from the perspective of both active measures and passive defence measures, with a view to contributing to the improvement of the legal system for the protection of TCM prescriptions.

## 2 Classification and characteristics of chinese medicine prescriptions

Chinese medicine prescriptions can be broadly classified into ancient classical prescriptions, veteran practitioners’ prescriptions, and folk prescriptions, which overlap with each other to some extent, but their respective characteristics are also quiet distinct. In managing different types of prescriptions, the most suitable strategies and approaches should be adopted based on their unique characteristics.

### 2.1 The ancient classical prescriptions (ACPs)

ACPs are researched, collected and collated by the government authorities, with the help of administrative power, and therefore have a strong ‘administrative’ color. The Law of the People’s Republic of China on Traditional Chinese Medicine (hereinafter referred to as the TCM Law) defines ACP as prescriptions recorded in ancient Chinese medical textbooks that are still widely used, have precise therapeutic effects, and possess obvious characteristics and advantages. Currently, the Chinese medicine authorities have published two batches of “Catalogue of Ancient Classical Famous Prescriptions”. In the second batch, in addition to prescriptions for Han Chinese medicine, prescriptions for Tibetan, Mongolian, Uyghur and Dai Chinese medicine have also been collected.

ACPs are the profound accumulation and precipitation of the Chinese nation’s splendid civilization of traditional Chinese medicine for thousands of years, and occupy an important position in the treasure trove of traditional Chinese medicine knowledge (Zhang et al., 2023). In the formulation of these classical prescriptions, the origin of each medicinal herb is precisely defined, which fundamentally ensures the reliability and stability of the herbal sources. At the same time, these prescriptions strictly adhere to specific processing techniques passed down from generation to generation, aiming to maximize the activation and preservation of the effective components of the medicines. Additionally, ACPs also specify the applicable conditions, contraindications, efficacy assessment criteria and withdrawal criteria, which have become highly mature and effective treatment protocols through the test of history (Yao et al., 2019). Formulations based on classic prescriptions, such as the *Liu Wei Di Huang Wan* series and the *Sheng Wei San* series, are still recognized as high-quality traditional Chinese medicines. Compared to other types of prescriptions, ACPs are widely circulated in book form, making their openness particularly prominent. While this characteristic has greatly facilitated the widespread dissemination and popularization of TCM knowledge, it has also made these precious knowledge resources vulnerable to external acquisition and utilization. In recent years, many ACPs have been spread overseas, developed into patented medicines by countries such as the United States, Japan, and Israel, and returned to the Chinese market (Gao and Long, 2013). This phenomenon not only demonstrates the value of ACPs in the global pharmaceutical field, but also serves as a wake-up call to the urgent need to strengthen the protection and inheritance of ACP to prevent further loss of this precious intellectual heritage.

### 2.2 Veteran practitioners’ prescriptions (VPPs)

VPPs originate from the inheritance and practice of renowned Chinese medicine practitioners, and China has carried out various initiatives focusing on the selection of renowned Chinese medicine practitioners. In 2010, the National Administration of Traditional Chinese Medicine (NATCM) released a list of renowned Chinese medicine practitioners and their studios. In 2016, the Ministry of Human Resources and Social Security and NATCM, and other units organized the evaluation and commendation of national TCM masters and renowned TCM practitioners nationwide, and the honorary title of ‘Renowned TCM Practitioner Nationwide’ would be awarded through a series of cumbersome procedures. In 2022, the NATCM, the National Health Commission, and other departments issued the ‘Opinions on Strengthening the Work of TCM talents in the New Era’, emphasizing the consolidation of the grassroots TCM talent pool and the continuous development of inheritance studios for renowned grassroots TCM practitioners. By 2025, 1–2 inheritance studios will be established for each county-level TCM medical institution. Up to now, China has conducted multiple rounds of evaluations for renowned TCM practitioners, and has repeatedly supplemented the list of renowned TCM practitioners and their studios, and issued corresponding results for achievement inspection, which has made an significant contribution to the qualification determination of renowned TCM practitioners and the development of the TCM industry.

VPPs refer to the relatively stable and clear prescriptions formed by the renowned practitioners in the inheritance studios or the practitioners who have been awarded the honourary title of ‘Renowned TCM practitioner Nationwide’ in the process of long-term clinical practice, which are the products of the combination of the theories of traditional Chinese medicine, the valuable experience of the former practitioners and the clinical practice, and they represent the high level of the academic and clinical skills of Chinese medicine practitioners at present (Zhang et al., 2019). Furthermore, VPPs serve as a crucial carrier for the inheritance and development of renowned TCM practitioner’s medical skills, principles and ethics. Specifically, medical skills embody the clinical wisdom and insights into disease diagnosis and treatment accumulated and refined over years of practice by renowned TCM practitioners, directly reflecting their superb medical expertise. Medical principles cover the core content of the TCM theoretical systems, serving as the cornerstone of the inheritance and the theoretical support of medical skills, and which encapsulates the academic insights and theoretical innovations systematically summarized by renowned TCM practitioners through long-term medical practice. As for medical ethics, it touches the deeper layers of academic thinking and cognitive thinking, representing the core soul and ultimate pursuit of inheritance, and demonstrates the profound insights and unique perspectives of renowned TCM practitioners (Zhang et al., 2024). Compared with ACP and folk prescriptions, the most prominent feature of VPPs is that the subject is clearer, making them more susceptible to protection under the existing intellectual property rights system.

### 2.3 Folk prescriptions (FPs)

FPs refer to prescriptions that have been circulating among the people and have certain clinical value although they have not undergone rigorous scientific verification. FPs originate from the long-term life practices of the Chinese nation and the accumulated experience in treating illnesses during the struggle against diseases (Pang, 2010), constituting an indispensable part of the traditional knowledge system of traditional Chinese medicine. Despite the view that FPs differ from ACPs and VPPs in that they lack scientific TCM theoretical guidance and sufficient clinical evidence-based research, their credibility and scientific efficacy are difficult to assess due to factors such as the absence of detailed written records or explanations, and geographical limitations in drug processing and material selection (Zhang et al., 2018). However, the value of FPs cannot be ignored. For example, the prescriptions recorded in Ge Hong’s ‘Zhou Hou Bei Ji Fang’ provided important inspiration for the discovery of artemisinin. In fact, throughout history, many Chinese medical practitioners have attached great importance to the collection and practice of FPs. For instance, Li Shizhen conducted in-depth learning among the people and traveled tirelessly across the country to compile ‘Ben Cao Gang Mu’. Many ACPs and VPPs originally derived from FPs, and through continuous refinement, innovation, and development by medical practitioners over generations, they gradually evolved into well-known prescriptions.

Compared to other forms of prescriptions, FPs are renowned for their two primary characteristics: dispersion and uncertainty. FPs are widely circulated among the populace and collected and compiled by various entities. Some are even passed down through master-apprentice relationships or within families from parents to children, and are often held by folk practitioners or itinerant practitioners as secret techniques for earning a living, not to be shared externally (Tian and Tian, 2014). The uncertainty of FPs mainly stems from the lack of rigorous scientific validation and systematic quality evaluation standards. The compositions of these prescriptions are not constrained by conventional norms, exhibiting a high degree of freedom and flexibility. This characteristic often renders FPs uniquely effective for certain endemic diseases and complex, difficult-to-treat conditions. When used appropriately, they can achieve remarkable therapeutic effects, but conversely, they may also yield adverse outcomes. Precisely because of this, the dispersed nature and uncertainty of FPs have long cast doubts on their safety and efficacy. In terms of inheritance, development, and legal protection, FPs face numerous challenges, which not only lead to confusion in their development, utilization, and management but also make this valuable resource a target coveted by large pharmaceutical companies in other countries, exacerbating the issue of resource loss.

## 3 Current status of protection for tcm prescriptions

### 3.1 Extraterritorial status quo

With the deepening of economic globalization, the value of TCM has become more and more prominent, coupled with the occurrence of the risk of infringement or even disappearance of TCM due to unfavourable protection and preservation of TCM. Based on this, many countries have begun to research and explore the preservation and transmission of traditional medicinal knowledge.

#### 3.1.1 India

India boasts a long-standing healthcare system, possessing one of the oldest medical systems in the world, and places great emphasis on the preservation and development of traditional medicine. In order to safeguard this invaluable traditional medical knowledge from misuse, the Indian government has taken a series of measures to strengthen its protection. Firstly, in accordance with the requirements of the Convention on Biological Diversity (CBD), India has set up the National Biodiversity Authority (NBA), specifically tasked with managing and protecting its genetic resources and related knowledge within its territory. The establishment of the NBA was particularly important in the face of repeated instances of plagiarism of traditional knowledge related to biogenetic resources by developed countries since the 1990s, such as the Basamati case, the Neem case, and the Turmeric case. (Shiva, 2024) Nowadays, any country seeking to access its genetic resources and related knowledge within India has to undergo the strict approval by NBA, which penalizes violations in accordance with the law. Secondly, India has also established the Traditional Knowledge Digital Library (TKDL), which is responsible for the systematic organization and compilation of indigenous medicinal knowledge, but is only accessible to specific entities for convenient patent examiner searches. The combination of knowledge disclosure and national protection effectively reduces the risk of traditional medicine knowledge being preemptively registered by other countries (Gupta, 2011). Through this way, India has successfully invalidated the US patent US5401504 for “turmeric” granted by the United States Patent and Trademark Office, as well as the European patent EP436257 for “Neem” (Claudia Finetti, 2011). Finally, India has also innovatively established the Bee Database, which is essentially a database of traditional medicine knowledge. The database covers the theoretical knowledge and practical experience of traditional medicine, such as formulas and therapies, available for public enquiry and use (Li and Wang, 2024). It is evident that India has taken numerous effective measures to protect traditional medicinal knowledge, not only ensuring its legitimate inheritance and development domestically but also earning it the respect and status it deserves on the international stage.

#### 3.1.2 Thailand

Thailand has always placed a high degree of importance on the protection and inheritance of traditional medicinal knowledge. To safeguard this invaluable legacy through specific legislation, Thai government enacted the ‘Act on the Protection and Promotion of Traditional Thai Medicinal Intelligence’. Section 16 of this act provides a particularly detailed classification of prescriptions, categorizing them into national formulas, general formulas, and personal formulas. National formulas, as officially declared by the Thai authorities, carry special medical and public health value and represent a unique and precious national resource. The production and development of such formulas must undergo rigorous government approval to ensure their legality and safety. General formulas, on the other hand, originate from Thailand’s ancient wisdom and have been widely circulated through generations. Due to their popularity and public nature, these formulas can be freely used by any entity without additional authorization. They serve as a testament to the inheritance and development of Thai traditional medicine over the course of history (Du, 2004). Personal formulas represent the culmination of individual exploration and research conducted during long-term medical practice. These formulas are highly personalized and unique and cannot be used by other entities without permission. When the holder is also the inventor, improper or inheritor of these formulas, they also have the right to apply for intellectual property protection to safeguard their rights and interests. Furthermore, Thailand has also formulated the ‘Traditional Thai Medicine Intellectual Property Management and Promotion Act’, which provides a detailed explanations regarding the registration model, protection rules, and the establishment of a dedicated management agency for traditional medicine, playing a crucial role in the protection and rational utilization of traditional medicinal knowledge (Sheng and Liu, 2024).

#### 3.1.3 Japan

Japan protects its traditional medicines primarily through a patent system. In the early days, Japan only granted patent protection to methods of manufacturing medicines, and it was not until 1976 that it started to implement comprehensive patent protection for medicines, and quickly constructed a set of perfect protection system. Within this system, the patent network strategy has become one of the main means for Japanese pharmaceutical companies. Specifically, the basic patent represents a core technology, while the peripheral patents encompass related technologies surrounding the core. The Japan’s patent system allows patent applications with only a single claim, enabling domestic enterprises to swiftly seize opportunities around the basic patent and apply for various distinctive peripheral patents, such as improved patents and application patents. In this way, Japanese enterprises have constructed a tight patent network, which not only effectively prevents competitors from bypassing or circumventing their patents but also forms an encirclement around the basic patents of the opponents, thus forcing the opponents to obtain their peripheral patents by exchanging their basic patents (He et al., 2006). Additionally, Japan has established a series of databases to inherit and develop traditional medicines. For example, the Japanese Pharmacopoeia sets important medicine standards for pharmaceutical researchers to reference and understand industry trends and developments. The Pharmaceuticals and Medical Devices Agency (PMDA) Database contains basic information on medicine prescriptions and clinical information, which is of great significance for the development and utilization of traditional medicine knowledge.

### 3.2 Domestic status quo

Currently, China’s protection of TCM prescriptions primarily relies on the intellectual property system (including the trade secret system) and the specialized regulation of the TCM law, jointly constructing a legal protective barrier. However, in practical operations, this protection system faces severe challenges from multiple aspects.

The protection provided by Copyright Law for TCM prescriptions is quite limited, mainly due to the fact that it is difficult for TCM prescriptions to constitute an ‘expression’ in the sense of the Copyright Law. For ACPs, as they originate from ancient TCM books, with a long history, they have long exceeded the copyright protection period. Similarly, the Copyright Law also offers limited protection for VPPs and FPs. Based on the principle of the dichotomy of idea and expression, if an ‘idea’ has only one or a very limited number of expressions in reality, then these expressions are also deemed as ‘idea’ and are not protected, which is known as the “Doctrine of Merger” (Zhu, 2018). Therefore, although VPPs and FPs are diverse in formulation, due to the emphasis on medical schools and theoretical foundations when prescribing, practitioners often only make adjustments to the composition and dosage for different physiques. Hence, it is difficult to meet the requirement of originality in expression, as one judgment pointed out that ‘the expressions of TCM prescriptions through prescriptions’ names, indications, ingredients, and preparation methods are common ways of expressing TCM prescriptions and do not constitute subject matter protected by the Copyright Law’. Therefore, as far as traditional Chinese medicine prescriptions are concerned, the Copyright Law can only provide a certain level of protection to documents, works, papers, and other materials derived from the prescriptions under specific conditions. It can not offer substantial protection for the prescriptions themselves and merely serves as an additional advantage rather than a fundamental safeguard.

The method of trade secret protection has the disadvantage that it is difficult to recover the loss once it is disclosed, and it also hinders the communication and development of prescription to a certain extent. Currently, the legal protection mechanism for trade secrets is the most frequently applied in the field of protecting TCM prescriptions, thanks to its advantages such as no administrative approval required, unlimited protection period, and relatively lower risk of leakage. For ACPs, even though they have entered the public domain, the derivative research and development achievements made by relevant institutions based on these prescriptions can still be protected under trade secret law. As for VPPS, it has condensed the wisdom crystallization of the renown practitioners’ long-term practice, which not only shows high value in clinical practice, but also contains huge commercial potential, and its secrecy is self-evident. Regarding FPs, many are passed down through generations within families or through teacher-apprentice relationships, with ‘not being disclosed externally’ being a crucial requirement in the process of inheritance, through the protection of trade secrets aligns well with the unique nature of these prescriptions (Zhao, 2024). However, while it is easy to keep secrets, it is also easy to lose them. Once information is disclosed, its value is lost and cannot be restored. In cases where confidentiality is compromised or threatened, the legal remedies available to holders of traditionally secret knowledge are typically limited to claims for damages or injunctions to prevent disclosure. In addition, the trade secret protection method is not conducive to the sharing of prescriptions. If all prescription-holding entities choose to adopt the trade secret method of protection, it will have a huge adverse impact on the inheritance, development and innovation of prescriptions.Therefore, the above drawbacks of protecting traditional medical knowledge through confidentiality determine that the method of trade secrets protection can only serve as a supplementary protection method, rather than the primary one (Han, 2021).

The Patent Law plays a pivotal role in the protection of TCM prescriptions, but its requirements are so demanding that it is difficult for TCM prescriptions to meet the requirements for patent applications.According to Article 25 of China’s Patent Law, methods for the diagnosis or treatment of diseases can not be patented. This legal provision necessitates that TCM prescriptions seek patent protection in the form of Chinese medicine preparations and other related products, but it is difficult for Chinese medicine preparations to meet the requirements of ‘novelty’ and ‘inventiveness’ of patents. The root of the problem lies in the fact that the three requirements emphasized in the patent law are particularly stringent for the special field of Chinese medicine. The patent law system originates from developed countries or regions, and its design concepts and evaluation criteria are often incompatible with the inherent characteristic and drug use laws of traditional Chinese medicine prescriptions. Western medicines are easy to pass patent examination because they have a clear chemical molecular structure, are more targeted, and the differences between drugs and their effects can be more accurately and objectively described and quantified. In contrast, TCM is guided by the theories of traditional Chinese medicine that have been continuously summarized through clinical practice by people of various ethnic groups over thousands of years. Without the guidance of these theories, it cannot be classified as TCM. Therefore, the mechanism of action of TCM puts more emphasis on the holistic nature, and the exertion of medicinal effects usually requires the combined effects of a variety of ingredients, rather than the simple superposition of chemical components of a single drug, which is not only difficult to separate, but also difficult to determine the exact active substance. However, it is precisely the profoundness and diversity of TCM theories that lead to different understandings and applications of TCM theories among different entities engaged in TCM inventions and creations. This essentially reflects the difference between the “reductionism based on quantitative analysis” of Western medicine and the “holistic view of the human body based on qualitative analysis” of TCM (Tao and Zhao, 2023). Therefore, how to protect Chinese medicine prescriptions in a more scientific and reasonable manner under the existing patent law framework, respecting the uniqueness of Chinese medicine while taking into account the general requirements of the patent law, has become an important issue that needs to be resolved urgently.

Furthermore, in the special legislation for the protection of Chinese medicine, there are problems such as unclear interpretation of provisions, which can only provide limited protection for Chinese medicine prescriptions. Specifically, in the field of TCM protection, China not only relies on the important pathway of the intellectual property rights system but also enacted the TCM Law in 2017. This law establishes a comprehensive and three-dimensional institutional protection framework for traditional knowledge of Chinese medicine from a variety of dimensions, such as TCM services, protection and development of traditional Chinese medicine, cultivation of TCM talents, scientific research on TCM, and inheritance and cultural dissemination of TCM. Among these, Article 43 explicitly stipulates that holders of traditional Chinese medicine knowledge shall have the right to inherit and use their held knowledge, as well as the rights to informed consent and benefit-sharing for others’ acquisition and utilization of their held traditional Chinese medicine knowledge. However, despite elevating the protection of traditional TCM knowledge to a high level, the law’s practical protection of traditional medicinal prescriptions remains relatively weak, because there is no specific explanation of what constitutes a ‘holder’ and the content of the rights, and the subject of the rights and the content of the rights are unclear (Ma and Zhan, 2024).

To sum up, both the intellectual property rights system and the specialized regulation of the TCM Law have certain limitations in protecting the legitimate rights and interests of TCM prescriptions, failing to provide comprehensive and effective safeguards. More critically, there is a lack of effective coordination and complementarity between these two pathways, failing to form a strong protective force. Therefore, there is an urgent need to optimize and adjust the protection system for TCM prescriptions, aiming to establish a more complete and efficient protection mechanism.

## 4 Improvement of the path of the prescription protection system of Chinese medicine

### 4.1 Passive defence measures

Graham Dutfield, a British intellectual property jurist, proposed the establishment of relevant database is one of the effective defensive protection mechanisms for traditional knowledge (Dutfield, 2003). However, there are still some problems in the establishment of Chinese medicine prescriptions database in China. The purpose of establishing such databases is based on two aspects: firstly, it is to comprehensively collect, systematically organize and digitally record traditional knowledge of Chinese medicine, represented by TCM prescriptions for permanent preservation; secondly, it is to prevent the improper and illegal acquisition, possession and utilization of TCM prescriptions to prevent the emergence of erroneous granting of patents, so as to better safeguard the interests of the right holders. Surveys indicate that approximately 2000 medical system-related patent application are granted annually India (Sharma, 2017). Up to now, the Indian Traditional Knowledge Digital Library has effectively prevented about 353 foreign countries from illegally granting patents related to traditional knowledge through the Traditional Knowledge Database. (See TKDL Outcomes against Bio, 2024) Currently, Article 30 of China’s TCM Law also clearly stipulates that the specific catalogue of ACPs shall be formulated by the State Administration of Traditional Chinese Medicine, in conjunction with the drug regulatory authority. In fact, since 2015, NATCM has established and released the Traditional Knowledge of TCM Database (TKTCMD), which is divided into five sub-databases: prescriptions, traditional diagnostic and therapeutic techniques, TCM medicine processing techniques, methods of health maintenance, and others. Up to now, TKTCMD has been released, including approximately 4000 prescriptions from prescription books before the Song and Yuan dynasties (He and Wang, 2019). However, although the purpose of establishing the database in China is quite clear, there are still major deficiencies in the collection methods and related supporting systems, which makes it difficult to comprehensively cover the ACPs, VPPs, and FPs.

The unique nature of each of the three types of prescriptions should be fully considered during the construction of the database. Firstly, from the perspective of collection mode, ACPs are significantly characterized by their public nature. At present, government departments are responsible for the collection, collation and preservation of ancient classical prescriptions, and should strive to achieve extensive coverage and detailed records. A completely open management model should be adopted for the ACPs to prevent biopiracy (Wu et al., 2020). Secondly, the scope of VPPs should be expanded appropriately for the collection of their experience prescriptions. Currently, the selection of renown Chinese medicine practitioners is primarily handled by the relevant government departments, but consideration could be given to delegating this selection authority to hospitals and other relevant units. Hospitals and other relevant units would directly decide on the recognition of renown Chinese medicine practitioners without the need to go through the cumbersome process of recommendation and declaration by the grassroots units. Government departments would mainly be responsible for setting the relevant standards and making the appropriate records. Once renown Chinese medicine practitioners are identified, their hospitals or scientific research institutions can take charge of collecting and registering their empirical prescriptions and promptly file them with government departments. This approach allows for the rapid aggregation of high-value empirical prescriptions from various hospitals and related units. Finally, the collection of FPs should be the responsibility of the administrative department of traditional Chinese medicine at the grassroots level. In the process of registration, the procedure for applying for utility model patents can be referenced, with only formal examinations conducted to review the completeness of documents, the standardization of formats, and the integrity of information, without substantive review. In the case of VPPs and FPs, instead, the right holders themselves decide whether to disclose them to the public. However, regardless of whether it is disclosed to the public or not, it should be disclosed to the patent examination department, in order to serve as a deterrent to improper patent applications. All in all, the collection of prescriptions should aim for comprehensive coverage and full registration, adopting targeted collection measures according to prescription types, striving to build a rich and reasonably classified database in the shortest possible time.

In the process of constructing the database, in addition to defining a fundamental and essential model structure, it is also necessary to adequately motivate the enthusiasm of registrants. This motivation can be achieved by clarifying the scope of the registrant’s rights. Specifically, Article 43 of the current TCM Law stipulates that the holder of TCM knowledge enjoys the rights to inherit and use such knowledge, give informed consent and share benefits. Therefore, for VPPs and FPs, all registrants of these prescriptions can be regarded as the holder and granted corresponding rights. Of course, resolving rights conflicts, where multiple holders claim rights to the same prescription, is necessary in this process. To address this issue, reference can be made to the protection mechanism of copyright law for works, which means that there is no need to dwell on the conflict of right holders at the time of initial registration but rather when infringement disputes arise, based on specific circumstances. Finally, for the realization of benefit sharing, the State Council’s TCM administrative department can establish a professional collective management organization responsible for formulating reasonable fee standards and a fair distribution mechanism, ensuring that every prescription holder receives their entitled benefit distribution, thereby further stimulating the enthusiasm of all parties to participate in registration and sharing.

### 4.2 Active improvement measures

Building a database can indeed play a pivotal role in defending against foreign entities utilizing TCM prescriptions for patent applications, thereby constructing a solid defence line for the intellectual property rights of TCM. However, solely relying on defensive measures is far from sufficient to achieve the goal of modernizing the development of TCM. To facilitate the inheritance and development of TCM, and help it go global to demonstrate strong competitiveness on the international stage, it is necessary to establish a relatively comprehensive legal environment for intellectual property rights as a safeguard. ‘Research on the Adaptability of the Characteristics of TCM Knowledge to Intellectual Property Protection’ is an important aspect of China’s efforts to strengthen the protection of intellectual property rights related to TCM. (Guidelines on Strengthening Intellectual Property, 2024).

In the legal system concerning intellectual property rights of traditional Chinese medicine, the patent system undoubtedly occupies a pivotal position in providing protection. However, given the unique characteristics of TCM, particularly its emphasis on holistic properties, it is challenging for TCM to meet the requirements of novelty, utility, and inventiveness that are currently emphasized in the patent system. The Doha Declaration, adopted in November 2001, made it clear that the granting of biotechnology patents should be examined in the context of respecting the specific interests of indigenous peoples and developing countries (Gibson, 2004). Currently, the examination standards concerning Chinese medicine patents are not determined, and there are no relevant provisions in international conventions. As a major applicant country for TCM invention patents, China can attempt to take the lead in establishing and implementing review standards for Chinese medicine invention patent applications. Firstly, regarding the novelty requirement, the standard for novelty should be broadened. In practical operations, if an individual dispenses drugs for others based on a prescription while retaining the prescription, although this constitutes use of the prescription, since others cannot ascertain its components and proportions solely through using the medication, such use should not be considered as public disclosure, and therefore, the prescription should not be deemed to have lost its novelty. Secondly, as far as utility is concerned, according to Article 22 of China’s Patent Law, it refers to the fact that an invention patent or utility model patent can be manufactured or used and can produce a positive effect. This provision implies a requirement for stable reproducibility of patents. However, for Chinese medicine, due to its emphasis on holism and the significant impact of herbal material quality on efficacy, the reproducibility requirement is somewhat harsh for TCM prescriptions (Fu et al., 2024). Therefore, for the examination of utility, the reproducibility requirement can be moderately lowered. From the perspective of intellectual property protection, for the utility of traditional medical knowledge, regardless of whether it is technically reproducible or economically viable on scale, if it can meet certain needs for disease treatment, prevention, and health maintenance, and provide intellectual assistance for the development of new drugs, it should be deemed to have utility (Yan et al., 2019). Finally, regarding the assessment of inventiveness, the requirement is that an invention must possess substantial characteristics and notable progress compared to prior art. For patent inventions based on prescriptions, this standard can be appropriately relaxed. Specifically, when a TCM invention developed based on a prescription can solve specific medical problems, produce unexpected therapeutic effects, or directly lead to commercial success, these factors can be considered comprehensively to determine that the invention is inventive. In summary, a patent provides exclusivity rights to the use and exploitation of inventions (Dietsch et al., 2008). China should boldly explore and establish patent examination standards suitable for the characteristics of TCM, so as to enhance its voice and influence on the international stage within the field of TCM. At the same time, this will also support Chinese entities in obtaining TCM patents and promote their widespread dissemination and application globally.

Additionally, in terms of active measures, China should also accelerate the standardization process of TCM. In today’s fierce competition of globalization, the deep integration of technology and standards has become the norm, and the right to formulate standards is often directly related to the initiative and voice in international economic trade. The establishment of TCM standardization hinges on balancing the relationship between standardization and personalization. TCM focuses on the careful assessment of individual cases and places great importance on the subjective feelings of patients, thus building a mindset centered on personalized diagnosis and treatment and evaluation of clinical practice. However, standards are the commonalities extracted from the intricacies of individuality, and will continue to be optimized and improved with the depth of individualized diagnosis and treatment practice. Therefore, the formulation of TCM standards needs to encourage the expression of individuality, taking full consideration and careful treatment of distinctive technologies and treatment protocols. This implies that standardization should not become a shackle that constrains personal experience and innovation. In short, although the academic heritage of Chinese medicine, clinical diagnosis and treatment and herbal concoctions technology all emphasize individual practice, experience and perception, and tend to personalized schemes and unique skills and diagnosis and treatment, standardization relies on the reproducibility and normality that are equally indispensable. Quality control and standardization of products are key to the ability of TCM to reach the international stage. The lack of international standards makes it difficult for quality to be widely recognized, and it is easy to be restricted by the quality standards of developed countries, thus hindering the internationalization of TCM. Therefore, China urgently needs to establish quality standards that conform to the development law of TCM and are accepted by the world, and at the same time combine them with the intellectual property rights of TCM, so as to greatly enhance the international status of TCM and promote the global dissemination of TCM culture.

## 5 Conclusion

The protection of TCM prescriptions necessitates a strategy that balances both active and passive measures, which are complementary and indispensable. The initiative such as establishing categorized TCM prescription databases can effectively mitigate the risk of other countries improperly utilizing our TCM prescriptions to apply for patents, thereby safeguarding our invaluable TCM cultural heritage from illegal infringement. In addition, at the level of positive measures, China should establish a multidimensional framework for the protection of intellectual property rights. Specifically, China should actively improve the patent system, while accelerating the standardisation process of Chinese medicine, facilitating the expansion of Chinese medicine enterprises in China, and enhancing China’s influence and discourse power in the field of Chinese medicine in the world; employing the copyright law to protect the individual prescription or the collection of prescriptions constituting a work; adopting the trademark system to protect the trademarks, font names and other logos of the Chinese medicine enterprises, which are able to embody their brand image and market competitiveness; and applying the trade secret system to provide long-term secret protection for the core technologies and preparation processes in Chinese medicine prescriptions. In summary, through the comprehensive application of active and passive measures, as well as legal systems such as copyright, trademark rights, and trade secrets, we can establish a comprehensive and multi-layered legal protection framework for the inheritance and development of TCM prescriptions in China (Xie, 2023). This will lay the foundation for the vigorous development of the TCM industry and ensure that the treasure of TCM prescriptions can be handed down from generation to generation, radiating new vitality and vigor.

## Data Availability

The original contributions presented in the study are included in the article/supplementary material, further inquiries can be directed to the corresponding author.
